# AI/ML driven prediction of COPD exacerbations and readmissions: a systematic review and meta-analysis

**DOI:** 10.3389/fdgth.2025.1641356

**Published:** 2025-12-18

**Authors:** Prajita Niraula, Mallika Upreti, Suman Kadariya, Bishal Poudel, Sujan Kadariya, Shreedhar Kunwar

**Affiliations:** 1 Independent Researcher, Kathmandu, Nepal; 2 Department of Computer Science, King’s College, Kathmandu, Nepal; 3 KIST Medical College and Teaching Hospital, Kathmandu, Nepal

**Keywords:** COPD exacerbation, hospital readmission, artificial intelligence, machine learning, meta-analysis, predictive modeling

## Abstract

**Background:**

Chronic obstructive pulmonary disease (COPD) exacerbations and hospital readmissions are major drivers of morbidity, mortality, and healthcare costs. Artificial intelligence and machine learning (AI/ML) approaches have been applied to predict these events, but their pooled performance and methodological rigor remain unclear.

**Methods:**

Following PRISMA 2020 guidelines, we conducted a systematic review and meta-analysis of peer-reviewed studies developing or validating AI/ML models for predicting acute exacerbations of COPD (AECOPD) or hospital readmissions. Databases (PubMed, IEEE Xplore, Cochrane Library, Semantic Scholar) were searched to 2025. Eligible designs included retrospective and prospective cohorts, randomized trials with embedded prediction, and case–control studies. Study quality was assessed using PROBAST, and evidence certainty with GRADE. Random-effects models pooled area under the ROC curve (AUC); subgroup analyses compared AECOPD vs. readmission outcomes and internal vs. external validation.

**Results:**

Thirteen studies were included, with sample sizes ranging from 110 to 113,786 patients. Most were retrospective cohorts using EHRs or claims data, while two used prospective or trial-based data. Models applied diverse algorithms, including random forests, gradient boosting, neural networks, and ensemble pipelines. The pooled AUC across all studies was 0.77 (95% CI: 0.74–0.80), with very high heterogeneity (I^2^ = 99.5%). Subgroup analyses showed similar performance for AECOPD prediction (AUC = 0.77; I^2^ = 98.9%) and readmission prediction (AUC = 0.73; I^2^ = 19.8%). Externally validated models (*n* = 4) achieved higher accuracy (AUC = 0.82) than internally validated models (AUC = 0.76), although differences were not statistically significant. Risk of bias was moderate to serious in 69% of studies, mainly due to incomplete reporting and overfitting.

**Conclusion:**

AI/ML models demonstrate moderate-to-high discriminatory accuracy in predicting COPD exacerbations and readmissions, with pooled AUCs of 0.73–0.77. However, high heterogeneity, limited external validation, and frequent methodological concerns restrict generalizability. Standardized reporting frameworks (TRIPOD-AI, PROBAST-AI), rigorous external validations, and prospective implementation studies are needed to translate these promising tools into clinical practice.

## Introduction

Chronic obstructive pulmonary disease (COPD) claims roughly 3.5 million lives each year, ranking as the world's fourth leading cause of death in 2021 and on course to become the third by 2030, according to the World Health Organization (1, 2). Each acute exacerbation (AECOPD), which is a sudden, sustained surge in respiratory symptoms, accelerates lung-function decline, triggers the majority of COPD-related hospital admissions, and drives much of the disease's estimated US$50 billion annual economic burden (3, 4). This recurring cycle of unpredictable exacerbations, frequent hospital readmissions costing over $15 billion annually, and escalating healthcare burdens poses a major challenge for clinicians and health systems, highlighting the urgent need for predictive, data-driven tools that can identify deterioration before it requires emergency intervention (4, 5).

In recent years, artificial intelligence (AI) and machine learning (ML) have emerged as powerful tools for enhancing risk prediction in chronic diseases such as COPD. These approaches enable the development of predictive models that integrate large, multimodal datasets to uncover complex patterns linked to clinical deterioration and hospital readmission. A growing body of studies has employed a wide spectrum of ML algorithms, ranging from traditional classifiers like decision trees and random forests to more sophisticated techniques such as gradient boosting machines (GBMs), deep neural networks, and hybrid fuzzy systems to forecastAECOPD or predict short-term readmission risk. In parallel, AI and ML have also been applied to other aspects of COPD care, including diagnosing COPD using CT images (6), oxygen therapy optimization through automated SpO₂ monitoring systems (7), and predicting long-term disease progression (8), demonstrating their broader utility across the disease management continuum. Model performance is typically evaluated using metrics such as the area under the receiver operating characteristic curve (AUC), accuracy, sensitivity, specificity, and F1 score, with varying levels of internal and external validation.

While individual studies underscore the promise of ML-based models in predicting COPD-related outcomes, the existing literature remains highly heterogeneous, varying substantially in population characteristics, data sources, modeling techniques, and outcome definitions. Critically, the comparative performance of these models across diverse clinical and methodological contexts has yet to be clearly established. To date, no comprehensive meta-analysis has systematically synthesized the diagnostic accuracy of AI/ML-driven models for predicting AECOPD or hospital readmissions using real-world or trial-based data.

Traditional diagnostic modalities [including radiography, polymerase chain reaction (PCR), and immunoassays] play a vital role in confirming COPD and identifying infectious triggers of exacerbations, but they have notable limitations when applied to prognosis and prediction. Radiographic and CT imaging can characterize disease severity and confirm diagnosis, and recent AI-assisted CT models have demonstrated high diagnostic accuracy (6), but these approaches remain largely reactive and do not forecast future exacerbation or readmission risk. PCR and immunoassays detect pathogens or inflammatory activity but are narrow in scope, failing to capture the multifactorial drivers of exacerbations such as comorbidities, environmental exposures, and longitudinal symptom trends. Major guidelines emphasize that no single biomarker or conventional test reliably predicts COPD exacerbations or readmissions (3). Systematic reviews further highlight that this persistent gap has motivated the development of AI/ML models aimed specifically at prognosis (8). Collectively, while indispensable for diagnosis and surveillance, traditional tools are insufficient for forecasting near-term deterioration. This prognostic gap creates a critical need for tools that can integrate multimodal data to dynamically assess risk, which is the precise capability offered by machine learning approaches.

This study addresses the identified gap by conducting a meta-analysis of peer-reviewed research on AI/ML models developed to predict COPD exacerbations and hospital readmissions. Our objectives are to quantify the pooled diagnostic accuracy of these models and to critically evaluate their methodological rigor and clinical applicability. By doing so, this review contributes to the growing body of evidence on the role of AI in respiratory medicine and provides guidance for future model development, validation, and implementation.

## Methods

### Eligibility criteria

This systematic review and meta-analysis included original, peer-reviewed studies reporting the development or validation of AI/ML-based models for predicting AECOPD or hospital readmissions in patients with confirmed COPD diagnoses. Eligible studies were required to include adult populations (≥18 years) with a primary diagnosis of COPD, utilize machine learning, deep learning, or hybrid AI approaches to predict either AECOPD or hospital readmission (regardless of timeframe), and report at least one quantitative model performance metric (e.g., AUC, accuracy, sensitivity, specificity, or F1 score. We included retrospective and prospective cohort studies, randomized trials with embedded predictive modeling components, and case-control studies.

Studies were excluded if they focused solely on mortality, diagnostic classification, or non-predictive associations; if they used only traditional statistical models without AI/ML components (such as logistic or Cox regression alone); or if they were reviews, editorials, conference abstracts without full results, or non-peer-reviewed preprints. Animal or *in vitro* studies were also excluded, as well as studies that lacked sufficient information to extract predictive performance metrics.

### Information sources and search strategy

A comprehensive literature search was conducted across electronic databases PubMed, IEEE Xplore, Semantic Scholar, and the Cochrane Library. These databases were searched from inception to 2025 using combinations of controlled vocabulary (e.g., MeSH terms) and free-text keywords related to COPD, machine learning, artificial intelligence, and predictive performance. Search strategies were customized per database, and filters were applied to limit results to English-language, human studies where applicable.

In addition to database searches, we manually screened the reference lists of included studies and relevant reviews, as well as related articles suggested by databases, to identify additional eligible records. All searches were restricted to English-language publications.

### Selection process

**Figure 1:**
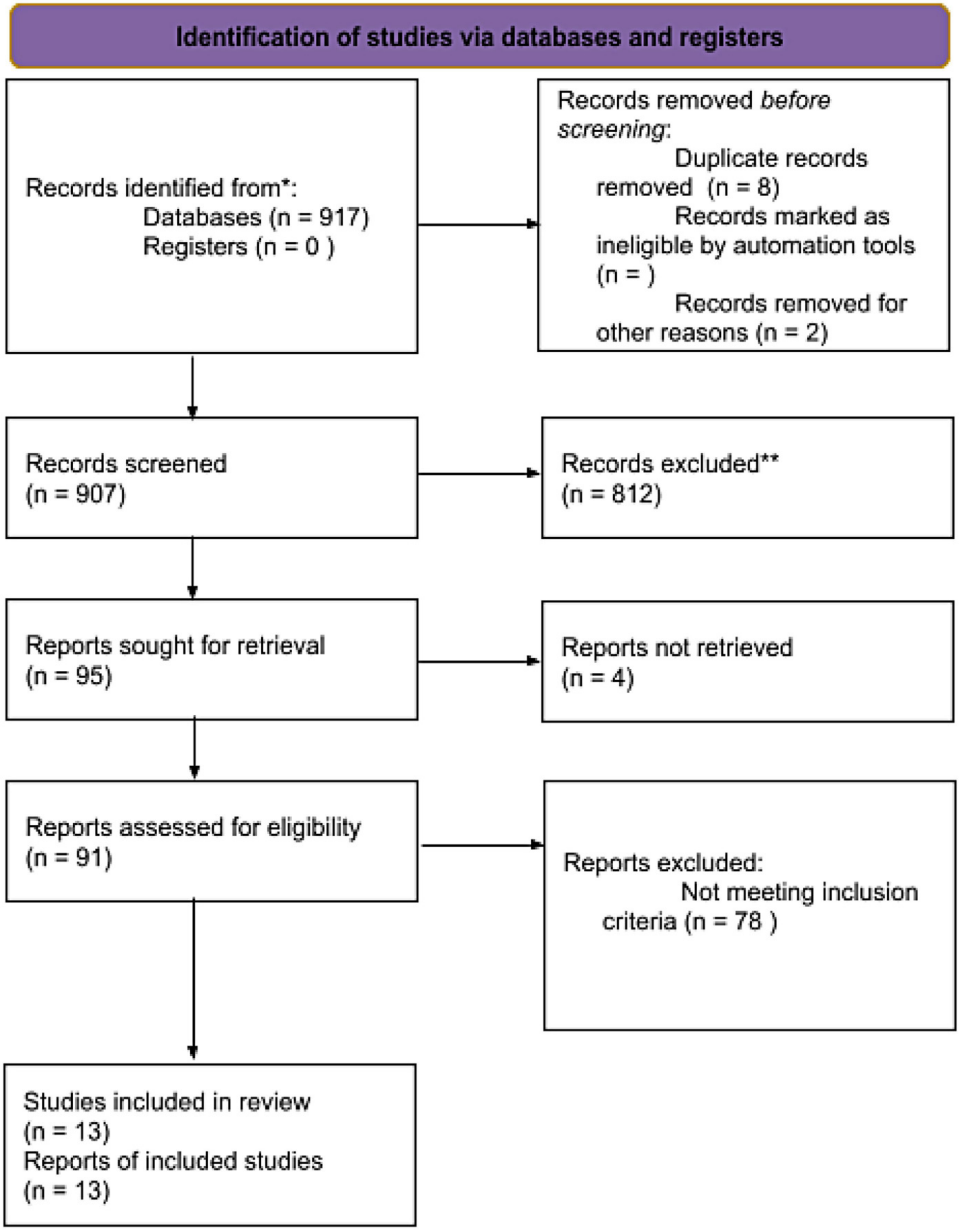
PRISMA flow diagram. PRISMA 2020 flow diagram summarizing the literature search and study selection process. The diagram shows the number of records identified through database searching, screened, assessed for eligibility, and ultimately included (*n* = 13 studies). Reasons for exclusion at the full-text stage are also detailed.

All retrieved titles and abstracts were screened independently by two reviewers. Full texts of potentially eligible studies were assessed against the inclusion criteria. Disagreements were resolved by discussion with a third reviewer. A PRISMA 2020 flow diagram (9) was used to document the study selection process (Figure 1). This study was not registered in PROSPERO because the project was conceived and completed retrospectively, with all methodological decisions finalized prior to data extraction and analysis. Although registration was not performed, we adhered to PRISMA 2020 guidelines to maximize transparency and reproducibility. The authors confirm that all methodological decisions were finalized prior to data extraction and analysis to minimize bias.

### Data collection process

Data were extracted using a structured form designed *a priori*, capturing study characteristics (authors, year), population details, outcome type (AECOPD vs. readmission), AI/ML model types, data sources, sample sizes, performance metrics (AUC, accuracy, sensitivity, specificity, F1 score), and validation strategies (e.g., internal cross-validation, external validation). Data extraction was performed in duplicate, and discrepancies were resolved via consensus.

### Data synthesis and meta-analysis

For studies reporting sufficient performance metrics, pooled estimates of AUC were synthesized using a random-effects meta-analysis model, given expected methodological heterogeneity (10). Sensitivity, specificity, and F1 scores were descriptively summarized due to inconsistencies in thresholding and reporting practices. Subgroup analyses were planned by outcome type (AECOPD vs. readmission), model class (e.g., tree-based, neural networks), and validation type (internal vs. external). Heterogeneity was assessed using the I^2^ statistic. All analyses were conducted in R.

We extracted information on how each primary study addressed missing data (e.g., multiple imputation, single imputation, or complete-case analysis) when reported. However, most studies did not explicitly describe their missing data handling strategy. No imputation was performed at the meta-analysis level; we synthesized only the reported results.

Because this is a meta-analysis rather than a single diagnostic study, we did not perform a traditional *a priori* power calculation. Instead, we followed recommended guidance for evidence syntheses by using a precision-based approach, aiming for a 95% CI half-width ≤0.05 around the pooled AUC. Our final synthesis of 13 studies (*n* ≈ 110–113,786 per study) achieved a pooled AUC of 0.77 with a 95% CI of 0.74–0.80, meeting this precision threshold. We also extracted whether primary studies reported their own sample size calculations, and deficiencies were noted in PROBAST risk-of-bias scoring.

## Results

### Study selection

A total of 917 records were identified through systematic searches of four electronic databases: PubMed, IEEE Xplore, Semantic Scholar, and the Cochrane Library. After removing 8 duplicate records and 2 that were excluded prior to screening, 907 records remained for title and abstract screening. Of these, 812 were excluded based on irrelevance to the review's population, intervention, or outcome criteria. The full texts of 95 records were sought for retrieval, of which 91 were successfully obtained and assessed for eligibility. Ultimately, 13 studies met all inclusion criteria and were included in the final qualitative synthesis and meta-analysis.

In addition to database searches, the reference lists of eligible studies and related articles were manually reviewed, contributing to the identification of some included studies. The selection process adhered to predefined eligibility criteria. Common reasons for exclusion during full-text review included use of traditional statistical models without AI or ML components, insufficient reporting of model performance metrics, study populations that included non-COPD or mixed respiratory cohorts, restricted behind pay-wall and non-original publication formats such as reviews, conference abstracts, or editorials. The full study selection process is summarized in the PRISMA 2020 flow diagram (11) (Figure 1).

### Study characteristics

Thirteen studies published between 2017 and 2025 were included in this meta-analysis. The majority employed retrospective cohort designs using real-world clinical or administrative datasets, while two studies (12, 13) incorporated prospective or trial-based data. Populations across studies consistently consisted of adults aged ≥18 years with confirmed COPD diagnoses. Outcomes targeted included AECOPD, both moderate and severe, and hospital readmissions, either all-cause or COPD-specific.

Machine learning approaches varied widely, encompassing tree-based methods (such as Random Forest, Gradient Boosting, XGBoost), support vector machines (SVM), neural networks, decision trees, and ensemble models. Logistic regression was used in some models for baseline comparison, though only studies with AI/ML-enhanced models were included. The highest-performing model in each study was retained for synthesis, based on reported performance metrics.

Model performance was most frequently assessed using the area under the receiver operating characteristic curve (AUC), followed by accuracy, sensitivity, specificity, and F1 score. Internal validation techniques included train-test splits and k-fold cross-validation; four studies additionally performed external validation on independent datasets, such as KNHANES, TORCH, or trial subsets. Sample sizes ranged from 110 participants (13) to over 100,000 records (14).

Data sources included electronic health records (EHRs), administrative health databases, digital health platforms, clinical registries, and wearable/telemonitoring systems. Most studies integrated multimodal data, combining physiological, demographic, environmental, and historical health variables to enhance predictive power. A tabular summary of study characteristics, including model types, target outcomes, validation strategies, and data sources, is provided in Table 1.

**Table 1:** Overview of study characteristics.

Study	Target outcome	Best model	AUC (95% CI)	Validation type	Sample size
Zeng et al. (14)	Severe AECOPD	Custom ML pipeline	0.866 (0.838–0.892)	Internal	43,576
Tavakoli et al. (15)	Hospitalized AECOPD	Gradient Boosting	0.82 (0.80–0.83)	External	108,433;
Bonomo et al. (16)	90-day readmission	XGBoost	0.73 [0.68–0.79]	Internal	3,238
Fakhraei et al. (17)	AECOPD admissions	Logistic Regression	0.77	Internal	64,609 admissions, 518 readmissions
Mohamed et al. (18)	30-day readmission	CHAID Tree	0.77	Internal	195
Jo et al. (19)	Moderate/severe AECOPD	MERF	0.721 (0.711–0.733)	Internal + External (KNHANES)	590
Shah et al. (13)	AECOPD prediction	RR + PR + SpO₂	0.682	Internal	110
Orchard et al. (20)	Admission risk	Neural Net	0.74 (0.67–0.80)	Internal	135
Kor et al. (21)	First-time AECOPD	GBM	0.836 (0.757–0.915)	Internal	
Lopez-Canay et al. (22)	90-day readmission	Ensemble Model	0.77	Internal	593
Safari et al. (12)	≥2 moderate/≥1 severe AECOPD	ACCEPT 2.0	0.756 (0.724–0.789)	External (TORCH)	1,803 (ECLIPSE), 1,091 (TORCH)
Jia et al. (23)	ICU admission/Mortality	Random Forest	0.828 (test)	Internal + test cohort	322
Peng et al. (24)	AECOPD aggravation	C5.0 Ensemble	0.803	Internal	410

**Table 2:** Meta-regression results: effect of study-level moderators on AUC.

Moderator	Comparison	β estimate	95% CI	*p*-value	I^2^ (%)	τ^2^
Outcome type	Readmission vs. AECOPD	−0.012	−0.077–0.054	0.73	97.22	0.0028
Validation strategy	Internal vs. External	−0.009	−0.075–0.058	0.8	99.5	0.0029
Combined model	Both moderators included	–	–	0.94	94.67	0.0031

**Table 3:** Certainty of evidence (GRADE summary).

Outcome	Risk of bias	Inconsistency	Indirectness	Imprecision	Publication bias	Overall certainty
AECOPD prediction	Moderate	Moderate	Low	Low	Some concerns	Moderate
Readmission prediction	Serious	Moderate	Low	Low	Some concerns	Moderate

### Risk of bias and applicability assessment

**Figure 2:**
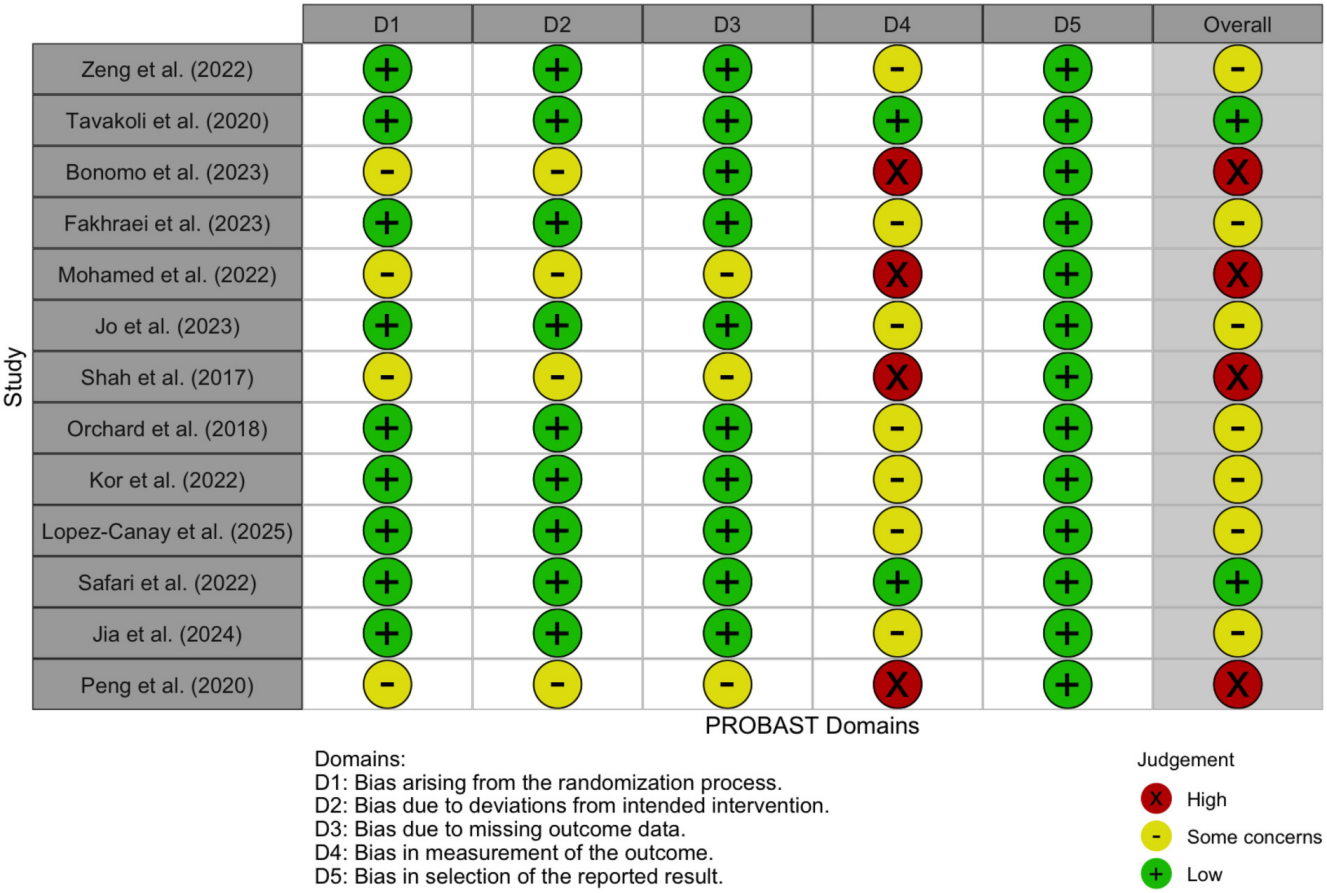
Illustrates domain-level and overall risk of bias judgments using a traffic light color scheme: green (+) indicates low risk, yellow (−) denotes some concerns, and red (×) reflects high risk.

Risk of bias was assessed using the PROBAST (Prediction model Risk Of Bias Assessment Tool), a validated framework specifically designed to evaluate studies that develop or validate multivariable prediction models (25). This tool evaluates methodological quality across four core domains: Participants, Predictors, Outcome, and Analysis. Each study was independently assessed by two reviewers and rated as having “Low”, “High”, or “Some concerns” for each domain. Overall risk of bias judgments were determined based on the aggregated domain-level ratings. Discrepancies were resolved through discussion, with adjudication by a third reviewer when necessary. To facilitate structured visualization, we implemented the assessment in R using the robvis package and generated a domain-level traffic light plot (Figure 2).

Most studies demonstrated low risk of bias in the domains of Participants, Predictors, and Outcome, indicating that study populations were appropriately defined, predictor variables were measured reliably, and outcomes were clearly defined and consistently assessed. However, the Analysis domain revealed notable variability and was the most frequent source of bias concerns. Common issues included insufficient handling or reporting of missing data, limited or absent external validation, inadequate adjustment for overfitting, and selective reporting of model performance metrics. Four studies [Bonomo et al. (16), Mohamed et al. (18), Shah et al. (13), and Peng et al. (24)] were judged to be at high overall risk of bias, primarily due to serious concerns in the Analysis domain. Conversely, studies such as Safari et al. (12) and Tavakoli et al. (14) were rated as having low overall risk, reflecting a more rigorous application of analytical methods and transparent reporting.

### Quantitative synthesis of results

All 13 included studies reported the area under the receiver operating characteristic curve (AUC) for their best-performing models in predicting either AECOPD or hospital readmissions. Individual AUC values ranged from 0.682 (13) to 0.866 (15), with the majority falling between 0.72 and 0.84. In several studies, confidence intervals were explicitly reported, allowing for greater reliability in variance estimation. For studies without reported intervals, standard errors were approximated based on test sample sizes using a binomial distribution assumption.

Additional performance metrics such as accuracy, sensitivity, specificity, and F1 score were reported in seven studies. However, heterogeneity in thresholds, outcome definitions, and validation methods precluded formal synthesis of these secondary metrics. For instance, Shah et al. (13) reported a sensitivity range of 60%–80%, but with an inverse trade-off in specificity (36%–68%), limiting comparability.

**Figure 3:**
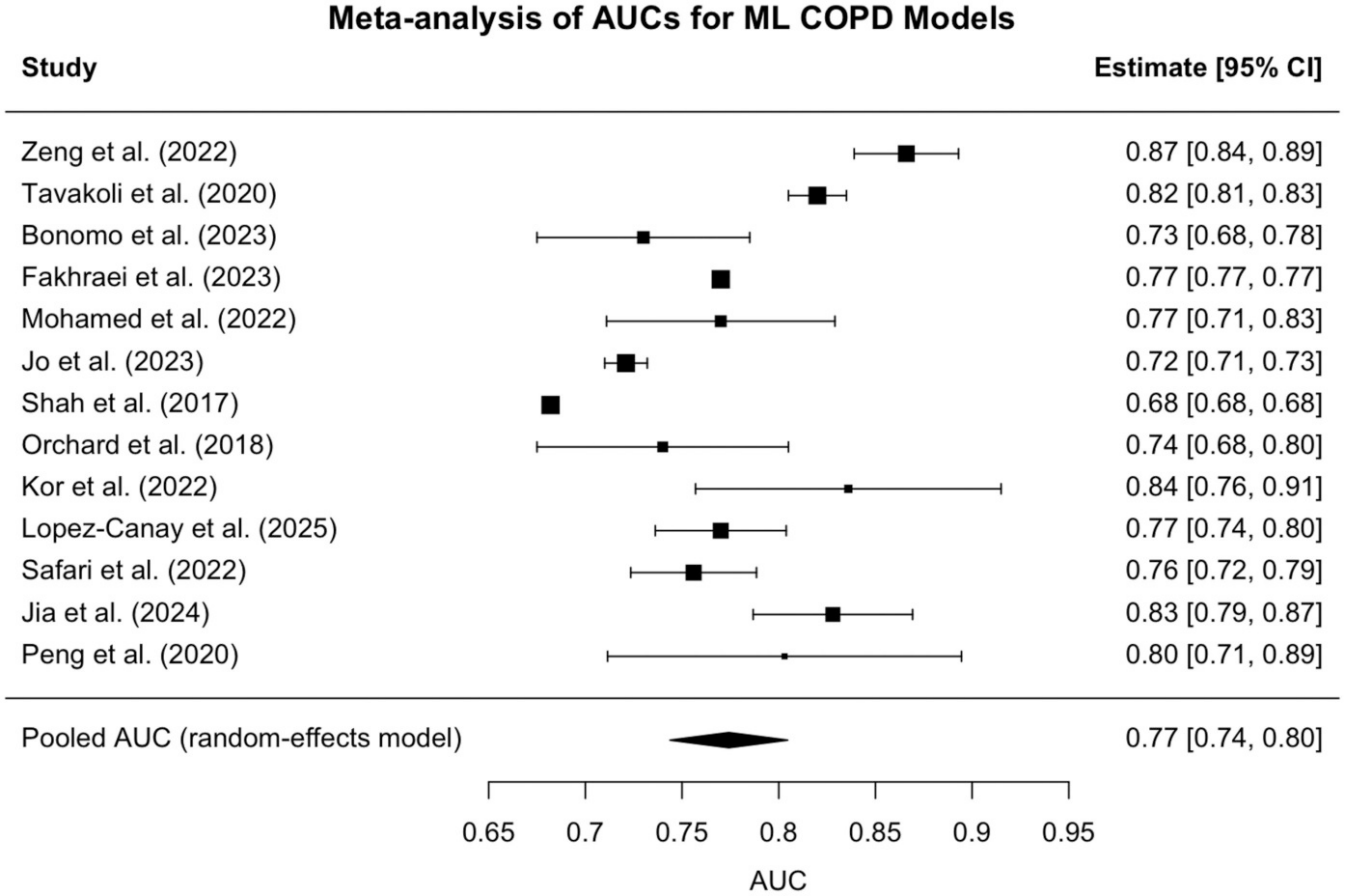
Forest plot - individual study AUCs. Forest plot of study-level AUCs for AI/ML models predicting COPD outcomes, where squares represent individual studies (sized by meta-analysis weight), horizontal lines show 95% confidence intervals, and the diamond indicates the pooled AUC (0.77, 95% CI: 0.74–0.80). AUCs ranged from 0.68 to 0.87 across studies, highlighting variability in performance but an overall moderate-to-high discriminatory accuracy for predicting acute exacerbations and hospital readmissions. All meta-analyses were conducted using a random-effects model with inverse variance weighting, and where 95% confidence intervals were not available, standard errors were approximated using the binomial distribution based on study sample size, with AUCs analyzed on the original scale unless otherwise noted.

A random-effects meta-analysis was conducted to synthesize the discriminative performance of the models based on AUC. The pooled AUC estimate across all 13 studies was 0.774 (95% CI: 0.744–0.805), indicating good overall discriminatory ability of machine learning models in this domain. However, between-study heterogeneity was substantial (I^2^ = 99.5%, *τ*^2^ = 0.0026), as confirmed by Cochran's *Q*-test [Q(12) = 3,415.83, *p* < 0.0001]. This heterogeneity likely reflects methodological variability across studies, including differences in sample sizes, predictor types, outcome definitions, and validation strategies (e.g., internal vs. external). A forest plot illustrating individual study AUCs and the pooled estimate is presented in Figure 3.

### Subgroup analysis: performance of models predicting AECOPD

We conducted a random-effects meta-analysis of studies focused specifically on the prediction of acute exacerbations of chronic obstructive pulmonary disease (AECOPD). This subgroup included 7 studies (12–15, 19–21) that reported the area under the receiver operating characteristic curve (AUC) as a measure of predictive performance.

The pooled estimate of AUC across studies was 0.77 [95% CI: 0.72–0.82], suggesting that machine learning-based models achieve a moderately high level of discrimination in identifying patients at risk of experiencing an AECOPD event.

**Figure 4:**
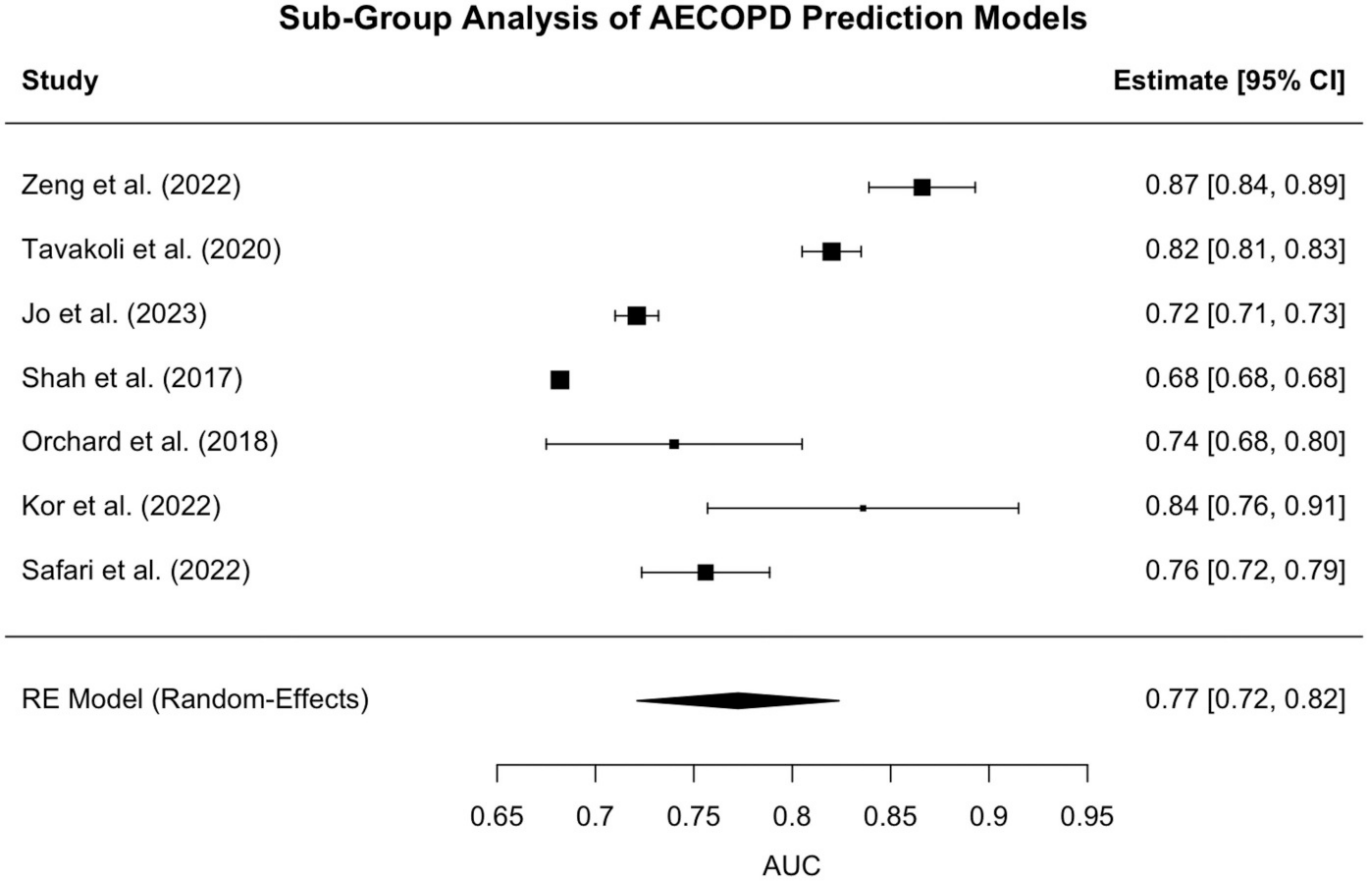
Forest plot for Sub-group analysis - AECOPD. Forest plot of individual study AUCs for AI/ML models predicting AECOPD. Squares represent study-level AUCs (sized by meta-analytic weight), horizontal lines indicate 95% confidence intervals, and the diamond denotes the pooled estimate (AUC 0.77, 95% CI: 0.72–0.82). Substantial heterogeneity was observed [I^2^ = 98.9%, Q(6) = 587.8, *p* < 0.0001], reflecting variation in study design, populations, and modeling strategies.

A forest plot of the included studies is shown in Figure 4, illustrating the variation in AUC estimates across models and the overall random-effects summary estimate.

Substantial heterogeneity was identified among the included studies. The estimated between-study variance (Tau^2^) was 0.0044 (standard error = 0.0028), indicating that a non-trivial portion of the variability in AUC estimates arises from differences across studies rather than random sampling error. The I^2^ statistic, which quantifies the proportion of total variation due to heterogeneity rather than chance, was 98.86%, signifying that nearly all variability in effect sizes is attributable to genuine differences between studies. Additionally, the Q-statistic was highly significant [Q(6) = 587.82, *p* < 0.0001], further confirming that the observed variation is unlikely to be due to chance alone. These findings highlight considerable inconsistency in model performance, likely driven by differences in data types, patient populations, predictor variables, and machine learning methodologies.

These results indicate significant variability in model performance across studies, likely due to differences in data sources (e.g., telemonitoring vs. administrative claims), feature sets, study populations, and modeling strategies.

Notably, Shah et al. (13) reported a remarkably narrow confidence interval [AUC = 0.68 (0.68, 0.68)], likely driven by an extremely large number of monitoring sessions. This artificially precise estimate may have disproportionately influenced the meta-analytic weight and should be interpreted with caution.

Despite the heterogeneity, the findings underscore the promise of predictive modeling for AECOPD detection and provide a benchmark for future model development and external validation efforts.

Jia et al. (23) was excluded from this subgroup meta-analysis because, although it targeted patients with AECOPD, its prediction outcome was ICU admission, reflecting in-hospital progression rather than the onset of exacerbation. While included in the overall meta-analysis because it used a machine learning model to predict a clinically significant deterioration outcome in a COPD population consistent with the study's inclusion criteria, it was excluded from subgroup pooling to preserve analytical clarity.

### Subgroup analysis: performance of models predicting hospital readmission

A random-effects meta-analysis was performed on four studies (16–18, 22) that evaluated machine learning models designed to predict hospital readmission among patients with COPD. The studies included various model architectures and sample sizes ranging from 195 to 3,238 patients. The pooled area under the receiver operating characteristic curve (AUC) was estimated at 0.73 [95% CI: 0.71–0.76], suggesting a moderate level of discriminatory performance across studies.

**Figure 5:**
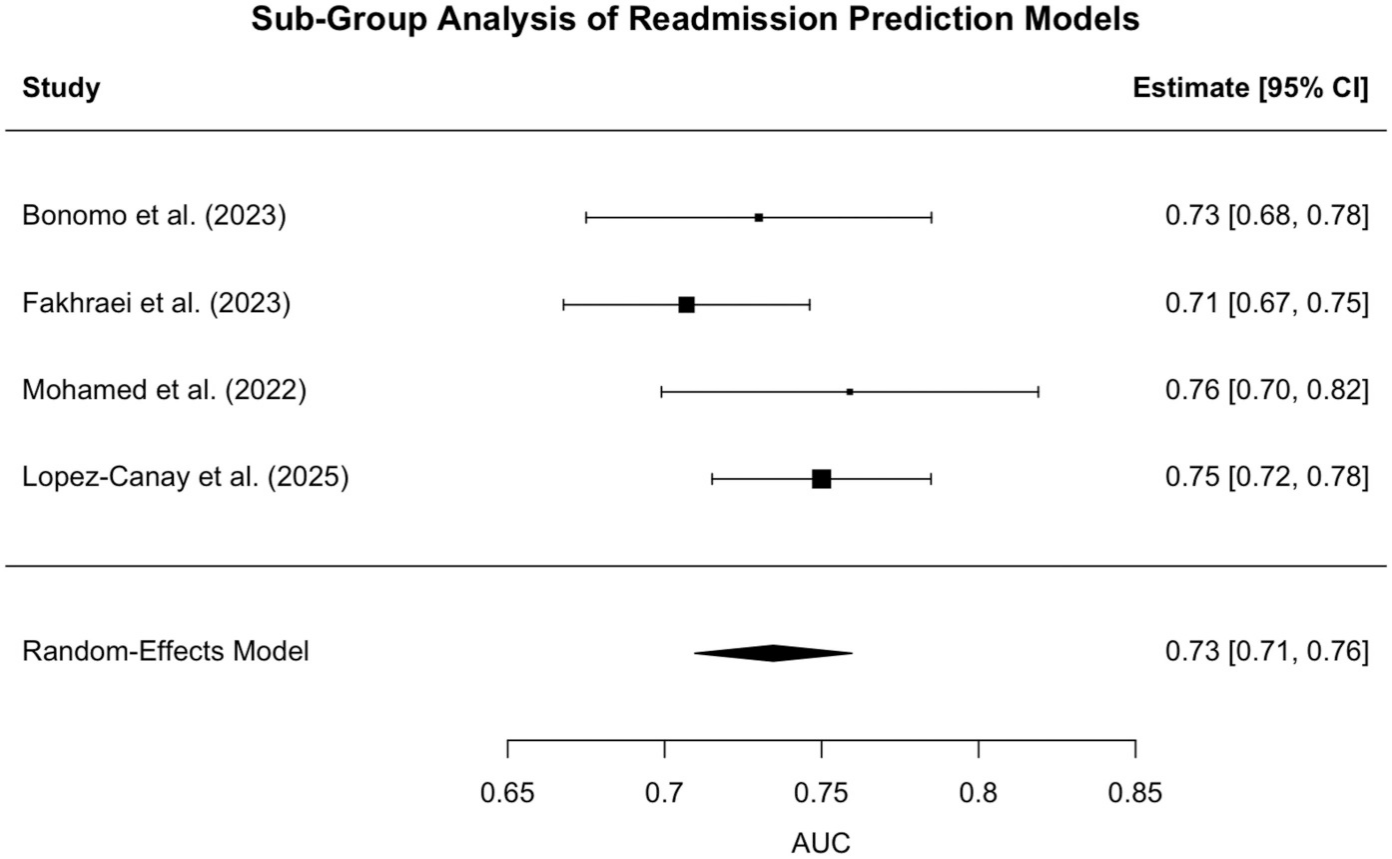
Forest plot for Sub-group analysis - readmission. Forest plot of individual study AUCs for AI/ML models predicting hospital readmissions in COPD. Squares represent study-level AUCs (sized by meta-analysis weight), horizontal lines indicate 95% confidence intervals, and the diamond denotes the pooled estimate (AUC 0.73, 95% CI: 0.71–0.76). Heterogeneity was low [I^2^ = 19.8%, Q(3) = 3.32, *p* = 0.35], suggesting consistent predictive performance across studies. However, this apparent low heterogeneity should be interpreted with caution, as the subgroup included only four studies. The limited number of comparisons reduces statistical power, and the observed consistency may reflect insufficient sample size rather than true homogeneity across studies.

A forest plot summarizing the individual study estimates and the overall pooled effect is presented in Figure 5.

Heterogeneity across the included studies was relatively low. The estimated between-study variance (Tau^2^) was 0.0001 (standard error = 0.0005), indicating minimal variability in AUC estimates attributable to differences between studies. The I^2^ statistic was calculated at 19.75%, suggesting that less than one-fifth of the total variability was due to true heterogeneity rather than sampling error. Furthermore, the Q-statistic was non-significant [Q(3) = 3.32, *p* = 0.3454], providing no evidence of statistically significant heterogeneity. These findings imply a high degree of consistency in the predictive performance of hospital readmission models across the included studies.

The non-significant *Q*-test and modest I^2^ value suggest that most of the variability in AUC values was likely attributable to random sampling error rather than true differences between studies. This contrasts with the high heterogeneity observed in the AECOPD prediction subgroup, indicating more consistency among readmission models in terms of performance.

Collectively, these findings indicate that while current machine learning models offer a modest and fairly consistent ability to predict COPD-related readmission risk, further refinement and external validation may be necessary to improve generalizability and real-world clinical utility.

### Subgroup AUC comparison

To determine whether predictive performance differed by clinical outcome type, we compared the AUC values of machine learning models designed to forecast AECOPD vs. those predicting hospital readmission. An independent two-sample *t*-test using Welch's correction for unequal variances was conducted. The mean AUC for AECOPD models (M = 0.774, SD ≈ 0.057) was slightly higher than that of readmission models (M = 0.737, SD ≈ 0.023), but this difference was not statistically significant [*t*(8.05) = 1.36, *p* = 0.21, 95% CI for the difference: −0.026–0.102].

These results suggest that although AECOPD-focused models showed a modest numerical advantage in discriminative performance, the observed difference is likely attributable to sampling variability and does not indicate a statistically meaningful performance gap between outcome types.

### Impact of validation strategy on model performance

To assess whether model validation strategy influenced reported performance, we compared AUC values between studies that used only internal validation (e.g., cross-validation, train/test splits) and those that included external validation on independent datasets. The mean AUC was 0.764 for internally validated models and 0.818 for externally validated models. However, this difference was not statistically significant [*t*(3.81) = –0.91, *p* = 0.42; 95% CI for the difference of −0.222–0.114].

These results suggest that while models evaluated on external datasets showed a slightly higher mean performance, the observed difference may be attributable to sampling variability. Moreover, the wide confidence interval and small number of externally validated studies underscore the need for more rigorous external evaluations before drawing strong conclusions about comparative performance.

### Meta-regression: outcome type and validation strategy

To confirm and extend these subgroup findings and further explore the sources of heterogeneity in reported model performance, we also conducted a meta-regression. Two study-level characteristics were examined as potential moderators: prediction target (AECOPD vs. hospital readmission) and validation strategy (internal-only vs. external validation). Neither factor was statistically associated with AUC. Specifically, models predicting readmission had slightly lower AUCs compared to those predicting AECOPD (*β* = –0.012, *p* = 0.73; 95% CI: −0.077–0.054), while studies using internal validation had marginally lower AUCs than those using external datasets (*β* = –0.009, *p* = 0.80; 95% CI: −0.075–0.058). When both moderators were included simultaneously, the model remained non-significant (*p* = 0.94), and none of the between-study variance was explained (R^2^ = 0%). Residual heterogeneity remained high across all models (I^2^ = 94.7% to 99.5%, *τ*^2^ ≈ 0.0028–0.0031), suggesting that differences in prediction target or validation strategy alone do not account for the wide variation in AUCs. These findings imply that other methodological factors such as feature engineering, sample composition, or model complexity may better explain the observed performance differences. The meta-regression results are summarized in Table 2.

### Certainty of evidence

The certainty of the pooled evidence was evaluated using a GRADE-adapted framework tailored to prediction model studies. Based on a synthesis of performance metrics and methodological quality across 13 included studies, the overall certainty of evidence is rated as moderate for both AECOPD and hospital readmission prediction models.

#### Risk of bias

Several studies exhibited moderate to serious risk of bias, primarily due to incomplete reporting, lack of pre-specified protocols, and missing data. While most studies clearly defined predictors and outcomes, only 4 out of 13 included external validation. The reliance on internal validation approaches likely inflated AUC estimates. As a result, the risk of bias was rated as moderate for AECOPD and serious for readmission models.

#### Inconsistency

Substantial heterogeneity was observed in AUC estimates:

- I^2^ = 98.86% for AECOPD,
- I^2^ = 19.75% for readmission,
- I^2^ = 99.5% overall.

Despite statistical heterogeneity, the direction of effect consistently favored ML model performance across all studies. Therefore, no downgrade was applied, although the inconsistency was rated moderate.

#### Indirectness

All studies were conducted in relevant populations (adults with COPD) and addressed appropriate outcomes. While definitions of AECOPD and data sources (e.g., EHR, claims, telemonitoring) varied, these differences did not compromise clinical applicability. No downgrade was applied.

#### Imprecision

The pooled AUC estimates demonstrated reasonably narrow confidence intervals:

- Overall: 0.774 [0.744–0.805]
- AECOPD: 0.77 [0.72–0.82]
- Readmission: 0.73 [0.71–0.76]

Most studies had sufficiently large sample sizes, and no downgrade for imprecision was warranted.

#### Publication bias

**Figure 6:**
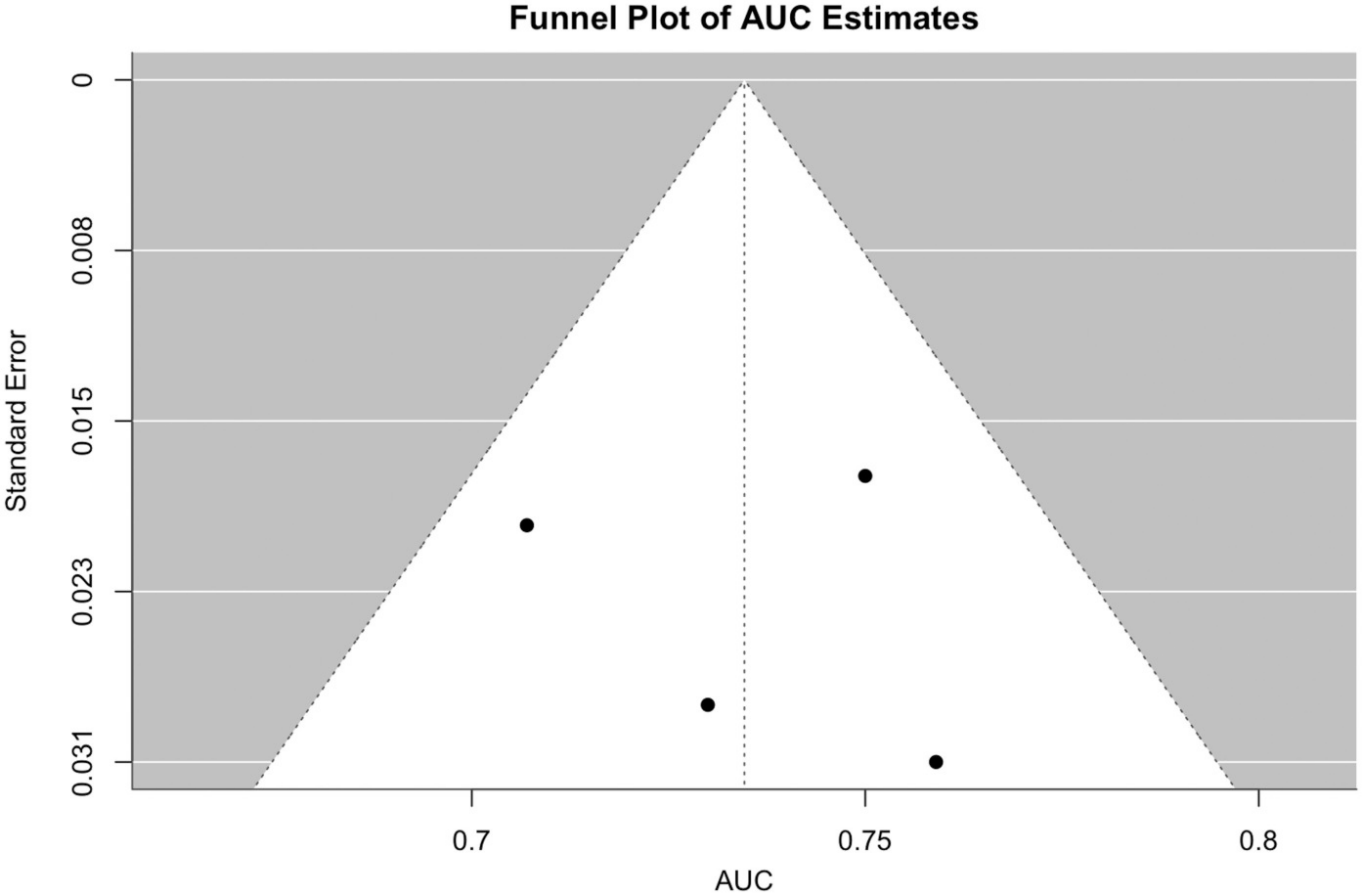
Funnel plot. Funnel plot of AUC estimates for AI/ML models predicting COPD outcomes. Each point represents an individual study plotted against its standard error; symmetry would suggest absence of publication bias.

Visual asymmetry in the funnel plot (Figure 6) suggested potential small-study effects. However, Egger's test was not statistically significant (*β* = 1.14, *p* = 0.32), providing no strong evidence of publication bias. Given limited external validation and variable reporting practices, some concerns remain, but these were not sufficient to justify a formal downgrade.

The GRADE certainty assessment for both outcomes is presented in Table 3.

The body of evidence supports the consistent, moderately precise, and clinically meaningful performance of machine learning models for predicting key COPD-related outcomes. Despite variability in study quality and methodology, the findings justify a moderate certainty rating, supporting cautious but optimistic consideration of these models in future clinical applications.

## Discussion

When contextualized against international benchmarks, the pooled discriminative performance observed in this review (overall AUC 0.77; AECOPD 0.77; readmission 0.73) is comparable to, and in some settings modestly higher than, established clinical scores and contemporary ML models. For 30-day readmissions, the LACE and HOSPITAL scores typically achieve AUCs of ∼0.73 and ∼0.69, respectively, in multi-condition cohorts (27), indicating that ML approaches in COPD are performing at least on par with widely used rules. For AECOPD risk, the recalibrated ACCEPT 2.0 tool reports AUCs in the ∼0.74–0.78 range across external cohorts, aligning closely with our AECOPD pooled estimate and underscoring that prospective, guideline-linked tools can reach similar discrimination (12). Beyond COPD, systematic and large-scale evaluations of heart failure and pneumonia readmission prediction frequently report ML AUCs around 0.70–0.80, situating our pooled readmission AUC at the upper bound of what is commonly achieved in chronic disease prognostics (26). These comparisons suggest that, while ML may not yet deliver transformative leaps in discrimination, current COPD models already meet or exceed the performance of entrenched clinical tools, with headroom for improvement via better calibration, external validation, and deployment studies.

Mechanistically, the higher and more variable accuracy in AECOPD prediction is biologically and operationally plausible. AECOPD events are often precipitated by airway inflammation, infectious triggers, exposure peaks, and prior exacerbation burden; signals that are increasingly captured in EHRs, pharmacy fills, telemonitoring streams, and time-varying features leveraged by ML. By contrast, hospital readmission reflects a broader mix of system-level and social determinants (post-discharge support, access, adherence, care coordination, comorbidity burden), which are only partially encoded in typical clinical datasets, plausibly explaining both the lower heterogeneity and the slightly lower pooled AUC in the readmission subgroup. From an implementation standpoint, this pattern implies that AECOPD models may benefit most from richer physiologic and environmental data streams and near-real-time monitoring, whereas readmission models may require integrated SDOH, utilization, and care-process features to meaningfully advance beyond current performance.

From a clinical perspective, ML-based AECOPD prediction models could be integrated into decision-support systems to identify high-risk patients for early intervention, potentially reducing emergency visits and hospitalizations. Readmission prediction models may complement existing discharge planning tools by targeting patients for closer post-discharge monitoring and transitional care. At present, these models should be viewed as adjunctive rather than replacement tools, pending further validation and demonstration of impact in prospective clinical trials.

Critically, our synthesis also highlights methodological ceilings that likely constrain apparent performance: (i) limited external validation in most primary studies; (ii) incomplete reporting of thresholds, calibration, and decision-analytic utility; and (iii) variable reference standards for defining AECOPD and readmissions, all of which can inflate optimism and hamper generalizability. These observations echo best-practice recommendations to pair discrimination with calibration curves, net benefit (decision curve analysis), and impact modeling, and to prioritize multisite external validation using harmonized outcomes and covariates (12, 27). Potential biases such as selection bias (non-representative cohorts), referral bias (more severe patients entering specialty datasets), and verification bias (inconsistent application of reference standards) may further limit generalizability and inflate reported accuracy.

In regards the limitations of this study, the reliance on reported AUCs without consistent reporting of confidence intervals introduced estimation uncertainty. The approximation of standard errors using binomial assumptions for studies lacking CIs may have introduced additional imprecision. Furthermore, although visual inspection of the funnel plot suggested asymmetry, Egger's test was not statistically significant (*p* = 0.32), providing no conclusive evidence of publication bias but leaving some concerns regarding selective reporting. We acknowledge that our analysiss studies. Another limitation of the included studies is the frequent lack of transparency regarding missing data handling. Many did not report whether imputation or complete-case analysis was applied, which introduces potential risk of bias. Because we relied on published summary data, we could not standardize or adjust for differences in missing data methods across studies did not involve a formal single-study power calculation; instead, we justified the adequacy of the pooled sample by demonstrating that the target precision of the pooled effect size was achieved. The lack of a uniform reference standard (e.g., variation between ICD-coded definitions vs. clinically adjudicated exacerbations) represents a source of potential spectrum bias and limits comparability. In addition, several design-related limitations should be noted. Many of the included studies relied on cross-sectional or retrospective designs, which preclude causal inference. A substantial proportion of models were developed using single-center data, limiting the generalizability of their performance across diverse care settings. Studies employing digital health or telemonitoring platforms may also be prone to self-selection bias, as participants who opt into monitoring programs are often healthier, more engaged, or differ systematically from the broader COPD population. Finally, the absence of external validation cohorts in most studies constrains confidence in the robustness of reported estimates.

## Conclusion

Machine learning models for COPD consistently demonstrated moderate-to-high discriminative accuracy, with pooled AUCs of 0.77 (95% CI 0.74–0.80) for AECOPD prediction and 0.73 (95% CI 0.71–0.76) for readmission models. Externally validated models (*n* = 4) achieved higher performance (AUC ∼0.82), underscoring the importance of validation beyond internal methods. However, heterogeneity was substantial for AECOPD (I^2^ = 98.9%) but low for readmission (I^2^ = 19.8%), indicating that performance varies widely depending on outcome type, data sources, and modeling strategies.

Despite promising accuracy, the evidence is constrained by methodological weaknesses, including overreliance on internal validation, inconsistent reference standards, incomplete reporting of calibration and decision-analytic measures, and frequent moderate-to-serious risk of bias on PROBAST assessment. Moreover, most studies used retrospective or single-center data, limiting generalizability, and did not clearly report handling of missing data.

Taken together, the evidence supports cautious but evidence-based optimism: ML models perform at least on par with widely used benchmarks such as the LACE (AUC ∼0.73) and HOSPITAL (AUC ∼0.69) scores, and align with prospective tools like ACCEPT 2.0 (AUC ∼0.74–0.78). However, further progress will require large-scale, multicenter external validations, transparent reporting (TRIPOD-AI, PROBAST-AI), and demonstration of real-world clinical utility and cost-effectiveness before routine integration into COPD care pathways.

## Data Availability

The original contributions presented in the study are included in the article/Supplementary Material, further inquiries can be directed to the corresponding author.
