# Supplementary material for: AI/ML driven prediction of COPD exacerbations and readmissions: a systematic review and meta-analysis
Source: Front Digit Health. 2025 Dec 18;7:1641356. doi: 10.3389/fdgth.2025.1641356 (PMC12756889; doi:10.3389/fdgth.2025.1641356)
Supplement: Supplementary file 1 [file Supplementaryfile1.pdf]

Supplementary Material A: Search Strategy and Study Selection Process

Supplementary Table 1: Summary of Databases Searched

Database	Records Found	Screened (Title/Abstract)	Full-Text Screened	Included Studies
PubMed	193	193	31	4
IEEE Xplore	54	54	4	1
Semantic Scholar	10	10	4	0
Cochrane Library	660	650	56	3
Reference Mining and Independent Search	—	—	—	5
Total	917	907	95	13

Search String and List of Included Studies

1.PubMed

("Pulmonary Disease, Chronic Obstructive"[Mesh] OR COPD[tiab] OR "chronic obstructive pulmonary disease"[tiab] OR "AECOPD"[tiab])
AND
("Artificial Intelligence"[Mesh] OR "Machine Learning"[Mesh] OR "Deep Learning"[Mesh] OR AI[tiab] OR "machine learning"[tiab] OR "predictive model"[tiab])
AND
("Patient Readmission"[Mesh] OR "Disease Progression"[Mesh] OR exacerbation[tiab] OR readmission[tiab] OR "risk prediction"[tiab])
AND
(English[lang] AND Humans[Filter])

- *Developing a Machine Learning Model to Predict Severe COPD Exacerbations* (JMIR)
- *Predicting Severe COPD Exacerbations* (AnnalsATS)

- *Predicting Likelihood and Cause of Readmission for COPD* (PubMed)
- *Multivariable Prediction Models for Risk of Readmission in COPD* (Tandfonline)

2. IEEE Xplore

Row 1:
"COPD" OR "chronic obstructive pulmonary disease" OR "AECOPD" OR "chronic obstructive lung disease"

AND
Row 2:
"artificial intelligence" OR "AI" OR "machine learning" OR "deep learning" OR
"neural network" OR "predictive model" OR "random forest" OR "support vector machine" OR
"supervised learning" OR "reinforcement learning" OR "predictive analytics"

AND
Row 3:
"exacerbation prediction" OR "readmission prediction" OR "hospital readmission" OR

"risk prediction" OR "prognostic model" OR "acute exacerbation" OR "clinical decision support"

Filter : Open-Access Only

- *Machine Learning Algorithms for COPD Readmission Prediction*

3. Semantic Scholar

(
("COPD" OR "chronic obstructive pulmonary disease" OR "AECOPD" OR "chronic obstructive lung disease")
AND
(
"machine learning" OR "deep learning" OR "artificial intelligence" OR "AI" OR
"neural network" OR "predictive model" OR "random forest" OR "XGBoost" OR

"support vector machine" OR "natural language processing" OR "NLP" OR
"time series analysis" OR "survival analysis"
)
AND
(
"exacerbation prediction" OR "hospital readmission" OR "readmission risk" OR
"acute exacerbation" OR "clinical deterioration" OR "prognostic model" OR
"risk stratification" OR "early warning system"
)
AND
(
"wearable" OR "telemonitoring" OR "remote monitoring" OR "telehealth" OR
"electronic health records" OR "EHR" OR "claims data" OR "spirometry" OR
"physiological signals" OR "multimodal data"
)
)

4. Cochrane Library

("COPD" OR "chronic obstructive pulmonary disease") AND ("machine learning" OR "ML" OR "artificial intelligence" OR "AI" OR "predictive model") AND ("exacerbation" OR "readmission")

- *Daily Predictive Model for COPD Exacerbation*
- *Prediction Using a Digital Health System*
- *ML for Telemonitoring-Based Risk Prediction*

5. Reference Mining and Independent Search

- *Explainable ML for First-Time Exacerbation in COPD*
- *Predicting COPD Readmission with Clinical Decision Support*

    - *ACCEPT 2.0 Tool Validation (Lancet)*
    - *ML-Based Models for ICU Admission and Mortality Prediction*
    - *Forecasting Aggravation Risk Using Clinical Indicators (Nature)*

Note on Excluded Studies

Full details of excluded articles and reasons for exclusion are available upon request. Common reasons included:

- No AI/ML methodology
- Focused on asthma or mixed populations
- Non-predictive or descriptive outcomes
- Access restrictions (paywalls)

Supplementary Material B: R code

```
library(metafor)
library(meta)
library(ggplot2)
library(reshape2)
library(robvis)
library(devtools)
library(robvis)
library(dplyr)
# -------------------------------------------------------


#PROBAST


# -------------------------------------------------------

library(robvis)
library(dplyr)

# -------------------------------------------------------
# PROBAST Data with Additional Domain Column
# -------------------------------------------------------
probast_data <- data.frame(
  Study = c(
    "Zeng et al. (2022)", "Tavakoli et al. (2020)",
    "Bonomo et al. (2023)", "Fakhraei et al. (2023)",
    "Mohamed et al. (2022)", "Jo et al. (2023)",
    "Shah et al. (2017)", "Orchard et al. (2018)",
    "Kor et al. (2022)", "Lopez-Canay et al. (2025)",
    "Safari et al. (2022)", "Jia et al. (2024)",
    "Peng et al. (2020)"
  ),
  D1 = c("Low", "Low", "Moderate", "Low", "Moderate", "Low",
                "Moderate", "Low", "Low", "Low", "Low", "Low", "Moderate"),
  D2= c("Low", "Low", "Moderate", "Low", "Moderate", "Low",
              "Moderate", "Low", "Low", "Low", "Low", "Low", "Moderate"),
  D3 = c("Low", "Low", "Low", "Low", "Moderate", "Low",
           "Moderate", "Low", "Low", "Low", "Low", "Low", "Moderate"),
```

```
  D4 = c("Moderate", "Low", "Serious", "Moderate", "Serious",
             "Moderate", "Serious", "Moderate", "Moderate", "Moderate",
             "Low", "Moderate", "Serious"),
  D5 = rep("Low", 13),
  Overall = c("Moderate", "Low", "Serious", "Moderate", "Serious",
            "Moderate", "Serious", "Moderate", "Moderate", "Moderate",
            "Low", "Moderate", "Serious")
)

# --------------------------------------------------------
# Convert to ROB2-compatible format
# --------------------------------------------------------
probast_robvis <- probast_data %>%
   mutate(across(c(D1, D2, D3, D4, D5, Overall),
              ~ case_when(
                . == "Low" ~ "Low",
                . == "Moderate" ~ "Some concerns",
                . == "Serious" ~ "High"
              )))

# --------------------------------------------------------
# Create Plot with Domain Adjustments
# --------------------------------------------------------
plot1 <- rob_traffic_light(
  data = probast_robvis,
  tool = "ROB2",
  colour = "cochrane"
) +
  labs(x = "PROBAST Domains") +
  scale_x_discrete(labels = c(
    "D1" = "Participants",
    "D2" = "Predictors",
    "D3" = "Outcome",
    "D4" = "Analysis",
    "D5" = "",  # Hide dummy domain label
    "Overall" = "Overall"
  )) +
  theme(axis.text.x = element_text(size = 10))

print
```

```
# --------------------------------------------------------

#Pooled AUC -- All studies

# --------------------------------------------------------
auc_data <- data.frame(
  study = c("Zeng et al. (2022)", "Tavakoli et al. (2020)", "Bonomo et al. (2023)", "Fakhraei et al. (2023)",
            "Mohamed et al. (2022)", "Jo et al. (2023)", "Shah et al. (2017)", "Orchard et al. (2018)",
            "Kor et al. (2022)", "Lopez-Canay et al. (2025)", "Safari et al. (2022)", "Jia et al. (2024)", "Peng et al. (2020)"),
  auc = c(0.866, 0.82, 0.73, 0.77, 0.77, 0.721, 0.682, 0.74, 0.836, 0.77, 0.756, 0.828, 0.803),
  ci_low = c(0.838, 0.80, 0.68, NA, NA, 0.711, 0.681, 0.67, 0.757, NA, 0.724, NA, 0.699),
  ci_high = c(0.892, 0.83, 0.79, NA, NA, 0.733, 0.682, 0.80, 0.915, NA, 0.789, NA, 0.882),
  sample_size = c(43576, 113786, 3238, 64609, 195, 590, 110, 135, 606, 593, 1091, 322, 410)
)

auc_data$sei <- with(auc_data, ifelse(
  !is.na(ci_low) & !is.na(ci_high),
  (ci_high - ci_low) / (2 * 1.96),
  sqrt((auc * (1 - auc)) / sample_size)  # Rough binomial approximation
))

res <- rma(yi = auc, sei = sei, data = auc_data, method = "REML")

summary(res)

forest(res, slab = auc_data$study,
       xlab = "AUC", mlab = "Pooled AUC (random-effects model)",
       main = "Meta-analysis of AUCs for ML COPD Models")

# --------------------------------------------------------

#Pooled AUC -- Sub-Group Analysis I : AECOPD Subgroup
```

```
# --------------------------------------------------------

data <- data.frame(
  Study = c("Zeng et al. (2022)", "Tavakoli et al. (2020)", "Jo et al. (2023)",
           "Shah et al. (2017)", "Orchard et al. (2018)", "Kor et al. (2022)",
           "Safari et al. (2022)"),
  AUC = c(0.866, 0.82, 0.721, 0.682, 0.74, 0.836, 0.756),
  Lower_CI = c(0.838, 0.80, 0.711, 0.681, 0.67, 0.757, 0.724),
  Upper_CI = c(0.892, 0.83, 0.733, 0.682, 0.80, 0.915, 0.789)
)


data$SE <- (data$Upper_CI - data$Lower_CI) / (2 * 1.96)


res <- rma(yi = AUC, sei = SE, data = data, method = "REML", slab = data$Study)


forest(res, xlab = "AUC", mlab = "RE Model (Random-Effects)",
      main = "Sub-Group Analysis of AECOPD Prediction Models")


# -------------------------------------------------------------------


#Pooled AUC -- Sub-Group Analysis II : Hospital Readmission Subgroup


# -------------------------------------------------------------------


data <- data.frame(
  Study = c("Bonomo et al. (2023)", "Fakhraei et al. (2023)", "Mohamed et al. (2022)",
"Lopez-Canay et al. (2025)"),
  AUC = c(0.73, 0.707, 0.759, 0.75),
  Lower_CI = c(0.68, NA, NA, NA),
  Upper_CI = c(0.79, NA, NA, NA),
  Sample_Size = c(3238, 518, 195, 593)
```

```
)

data$SE <- with(data, ifelse(
  is.na(Lower_CI),
  sqrt((AUC * (1 - AUC)) / Sample_Size),
  (Upper_CI - Lower_CI) / (2 * 1.96)
))

res <- rma(yi = AUC, sei = SE, data = data, method = "REML", slab = data$Study)

forest(res, xlab = "AUC", mlab = "Random-Effects Model",
       main = "Sub-Group Analysis of Readmission Prediction Models")

summary(res)

# ---------------------------------------------------------------------

#Pooled AUC -- t-test with Welch's correction to compare AUC
#values between AECOPD and readmission prediction subgroups

# ---------------------------------------------------------------------

aecopd_auc <- c(0.866, 0.82, 0.721, 0.682, 0.74, 0.836, 0.756)
readmission_auc <- c(0.73, 0.707, 0.759, 0.75)

t.test(aecopd_auc, readmission_auc, var.equal = FALSE)

# ---------------------------------------------------------------------

#AUC values for each group based on validation strategy
```

```
# ------------------------------------------------------------------

# Internal validation only
auc_internal <- c(
  0.866,  # Zeng et al.
  0.73,   # Bonomo et al.
  0.707,  # Fakhraei et al.
  0.759,  # Mohamed et al.
  0.682,  # Shah et al.
  0.74,   # Orchard et al.
  0.836,  # Kor et al.
  0.75,   # Lopez-Canay et al.
  0.803   # Peng et al.
)

# External validation included
auc_external <- c(
  0.82,   # Tavakoli et al.
  0.721,  # Jo et al.
  0.756,  # Safari et al.
  0.973   # Jia et al.
)

# Perform Welch's two-sample t-test
t.test(auc_internal, auc_external, var.equal = FALSE)

# -------------------------------------------------------
# Meta-Regression: Outcome Type and Validation Strategy
# -------------------------------------------------------


auc_data$outcome_type <- c("AECOPD", "AECOPD", "Readmission", "Readmission",
"Readmission",
                           "AECOPD", "AECOPD", "AECOPD", "AECOPD", "Readmission",
                           "AECOPD", "AECOPD", "Readmission")

auc_data$validation <- c("Internal", "External", "Internal", "Internal", "Internal",
                         "External", "Internal", "Internal", "Internal", "Internal",
                         "External", "External", "Internal")
```

```
auc_data$outcome_type <- factor(auc_data$outcome_type)
auc_data$validation <- factor(auc_data$validation)


res_outcome <- rma(yi = auc, sei = sei, mods = ~ outcome_type, data = auc_data, method =
"REML")
summary(res_outcome)


res_validation <- rma(yi = auc, sei = sei, mods = ~ validation, data = auc_data, method =
"REML")
summary(res_validation)


res_both <- rma(yi = auc, sei = sei, mods = ~ outcome_type + validation, data = auc_data,
method = "REML")
summary(res_both)

# -------------------------------------------------------
# Funnel Plot and Egger's Test for Publication Bias
# -------------------------------------------------------

# Funnel plot
funnel(res, main = "Funnel Plot of AUC Estimates", xlab = "AUC")

# Egger's test
egger_test <- rma(yi = auc, sei = sei, mods = ~ sei, data = auc_data, method = "REML")
summary(egger_test)
```

Supplementary Material C: PRISMA 2020 Checklist

Section and Topic	Item #	Checklist item	Location where item is reported
**TITLE**			
Title	1	Identify the report as a systematic review.	Title Page
**ABSTRACT**			
Abstract	2	See the PRISMA 2020 for Abstracts checklist.	
**INTRODUCTION**			
Rationale	3	Describe the rationale for the review in the context of existing knowledge.	Introduction
Objectives	4	Provide an explicit statement of the objective(s) or question(s) the review addresses.	Abstract & Introduction
**METHODS**			
Eligibility criteria	5	Specify the inclusion and exclusion criteria for the review and how studies were grouped for the syntheses.	Methods
Information sources	6	Specify all databases, registers, websites, organisations, reference lists and other sources searched or consulted to identify studies. Specify the date when each source was last searched or consulted.	Methods
Search strategy	7	Present the full search strategies for all databases, registers and websites, including any filters and limits used.	Appendix A
Selection process	8	Specify the methods used to decide whether a study met the inclusion criteria of the review, including how many reviewers screened each record and each report retrieved, whether they worked independently, and if applicable, details of automation tools used in the process.	Methods
Data collection process	9	Specify the methods used to collect data from reports, including how many reviewers collected data from each report, whether they worked independently, any processes for obtaining or confirming data from study investigators, and if applicable, details of automation tools used in the process.	Method
Data items	10a	List and define all outcomes for which data were sought. Specify whether all results that were compatible with each outcome domain in each study were sought (e.g. for all measures, time points, analyses), and if not, the methods used to decide which results to collect.	Results
	10b	List and define all other variables for which data were sought (e.g. participant and intervention characteristics, funding sources). Describe any assumptions made about any missing or unclear information.	Results
Study risk of bias assessment	11	Specify the methods used to assess risk of bias in the included studies, including details of the tool(s) used, how many reviewers assessed each study and whether they worked independently, and if applicable, details of automation tools used in the process.	Results
Effect measures	12	Specify for each outcome the effect measure(s) (e.g. risk ratio, mean difference) used in the synthesis or presentation of results.	Results
Synthesis methods	13a	Describe the processes used to decide which studies were eligible for each synthesis (e.g. tabulating the study intervention characteristics and comparing against the planned groups for each synthesis (item #5)).	Results
	13b	Describe any methods required to prepare the data for presentation or synthesis, such as handling of missing summary statistics, or data conversions.	Results
	13c	Describe any methods used to tabulate or visually display results of individual studies and syntheses.	Results
	13d	Describe any methods used to synthesize results and provide a rationale for the choice(s). If meta-analysis was performed, describe the model(s), method(s) to identify the presence and extent of statistical heterogeneity, and software package(s) used.	Results

Section and Topic	Item #	Checklist item	Location where item is reported
	13e	Describe any methods used to explore possible causes of heterogeneity among study results (e.g. subgroup analysis, meta-regression).	Results
	13f	Describe any sensitivity analyses conducted to assess robustness of the synthesized results.	
Reporting bias assessmen t	14	Describe any methods used to assess risk of bias due to missing results in a synthesis (arising from reporting biases).	Results
Certainty assessmen t	15	Describe any methods used to assess certainty (or confidence) in the body of evidence for an outcome.	Results
**RESULTS**			
Study selection	16a	Describe the results of the search and selection process, from the number of records identified in the search to the number of studies included in the review, ideally using a flow diagram.	Results
	16b	Cite studies that might appear to meet the inclusion criteria, but which were excluded, and explain why they were excluded.	Methods
Study characterist ics	17	Cite each included study and present its characteristics.	Results
Risk of bias in studies	18	Present assessments of risk of bias for each included study.	Results
Results of individual studies	19	For all outcomes, present, for each study: (a) summary statistics for each group (where appropriate) and (b) an effect estimate and its precision (e.g. confidence/credible interval), ideally using structured tables or plots.	Results
Results of syntheses	20a	For each synthesis, briefly summarise the characteristics and risk of bias among contributing studies.	Results
	20b	Present results of all statistical syntheses conducted. If meta-analysis was done, present for each the summary estimate and its precision (e.g. confidence/credible interval) and measures of statistical heterogeneity. If comparing groups, describe the direction of the effect.	Results
	20c	Present results of all investigations of possible causes of heterogeneity among study results.	Results
	20d	Present results of all sensitivity analyses conducted to assess the robustness of the synthesized results.	-
Reporting biases	21	Present assessments of risk of bias due to missing results (arising from reporting biases) for each synthesis assessed.	
Certainty of evidence	22	Present assessments of certainty (or confidence) in the body of evidence for each outcome assessed.	Results
**DISCUSSION**			
Discussion	23a	Provide a general interpretation of the results in the context of other evidence.	Results
	23b	Discuss any limitations of the evidence included in the review.	Results
	23c	Discuss any limitations of the review processes used.	Results
	23d	Discuss implications of the results for practice, policy, and future research.	Results
**OTHER INFORMATION**			
Registratio n and protocol	24a	Provide registration information for the review, including register name and registration number, or state that the review was not registered.	Methods
	24b	Indicate where the review protocol can be accessed, or state that a protocol was not prepared.	
	24c	Describe and explain any amendments to information provided at registration or	Methods

Section and Topic	Item #	Checklist item	Location where item is reported
		in the protocol.	
Support	25	Describe sources of financial or non-financial support for the review, and the role of the funders or sponsors in the review.	Title Page
Competing interests	26	Declare any competing interests of review authors.	Title Page
Availability of data, code and other materials	27	Report which of the following are publicly available and where they can be found: template data collection forms; data extracted from included studies; data used for all analyses; analytic code; any other materials used in the review.	Title Page

From: Page MJ, McKenzie JE, Bossuyt PM, Boutron I, Hoffmann TC, Mulrow CD, et al. The PRISMA 2020 statement: an updated guideline for reporting systematic reviews. BMJ 2021;372:n71. doi: 10.1136/bmj.n71. This work is licensed under CC BY 4.0. To view a copy of this license, visit https://creativecommons.org/licenses/by/4.0/
